# Post-fatigue fracture resistance of metal core crowns: press-on metal ceramic versus a conventional veneering system

**DOI:** 10.4317/jced.52267

**Published:** 2015-04-01

**Authors:** Mª Fernanda Solá-Ruiz, Rubén Agustín-Panadero, Carlos Campos-Estellés, Carlos Labaig-Rueda

**Affiliations:** 1 DDS, PhD, MD, Adjunct Lecturer, Department of Buccofacial Prosthetics. Faculty of Medicine and Dentistry, University of Valencia, Spain; 2 DDS, PhD, Associate Lecturer, Department of Buccofacial Prosthetics. Faculty of Medicine and Dentistry, University of Valencia, Spain; 3 DDS, Collaborator Lecturer, Department of Buccofacial Prosthetics. Faculty of Medicine and Dentistry, University of Valencia, Spain; 4 DDS, PhD, MD, Senior Lecturer, Department of Buccofacial Prosthetics. Faculty of Medicine and Dentistry, University of Valencia, Spain

## Abstract

**Background:**

The aim of this in vitro study was to compare the mechanical failure behavior and to analyze fracture characteristics of metal ceramic crowns with two veneering systems – press-on metal (PoM) ceramic versus a conventional veneering system – subjected to static compressive loading.

**Material and Methods:**

Forty-six crowns were constructed and divided into two groups according to porcelain veneer manufacture. Group A: 23 metal copings with porcelain IPS-InLine veneering (conventional metal ceramic). Group B: 23 metal copings with IPS-InLine PoM veneering porcelain. After 120,000 fatigue cycles, the crowns were axially loaded to the moment of fracture with a universal testing machine. The fractured specimens were examined under optical stereomicroscopy and scanning electron microscope.

**Results:**

Fracture resistance values showed statistically significant differences (Student’s t-test) regarding the type of ceramic veneering technique (p=0.001): Group A (conventional metal ceramics) obtained a mean fracture resistance of 1933.17 N, and Group B 1325.74N (Press-on metal ceramics). The most common type of fracture was adhesive failure (with metal exposure) (p=0.000). Veneer porcelain fractured on the occlusal surface following a radial pattern.

**Conclusions:**

Metal ceramic crowns made of IPS InLine or IPS InLine PoM ceramics with different laboratory techniques all achieved above-average values for clinical survival in the oral environment according to ISO 6872. Crowns made with IPS InLine by conventional technique resisted fracture an average of 45% more than IPS InLine PoM fabricated with the press-on technique.

** Key words:**Mechanical failure, conventional feldspathic, pressable ceramic, chewing simulator, thermocycling, compressive testing, fracture types, scanning electron microscope.

## Introduction

Metal ceramic crowns are a treatment that has been – and still is – in common use for prosthetic restorations supported by natural teeth or dental implants (1,2).

Although new ceramics such as zirconium oxide offer encouraging expectations in terms of strength and aesthetics, metal-ceramic restorations continue to be the treatment of choice in patients with parafunctional disorders and in posterior areas because of its high mechanical strength and predictability. These restorations enjoy a combination of strength and precision provided by the metal and aesthetics provided by the ceramic coating (3).

Metallic core restorations are usually processed with a conventional veneering system (conventional metal ceramic) but an alternative option is the use of the press-on metal ceramic technique – PoM (pressed-on-metal).

The conventional veneering system takes the metal coping, mechanically coating it with ceramic, which is bonded chemically; the chemical adhesive bond is achieved by sintering. The veneering procedure uses ceramic powders of porcelain composition of different color, mixed to achieve the finished restoration’s desired appearance (4).

The press-on metal system has been used successfully for almost two decades for fabricating complete ceramic restorations (3). It can also be used on restorations with metal cores (5,6). The metal coping is fabricated using the same technique but the ceramic veneer is made using a wax-up and a hot press furnace.

The combination of pressing and heat treatment provides a more uniform distribution of the leucite crystals in the glass matrix, providing the porcelain with greater strength (7).

Pressable ceramics are known to possess many desirable properties: the ceramic application technique is simpler and quicker than some of the conventional techniques available, provides acceptable marginal accuracy, and eliminates the need to compensate for the 20% shrinkage seen with traditional porcelain firing (8,9).

Fracture resistance is the deciding factor for determining the longevity of a restoration in the oral environment. Restorations possessing high fracture resistance have predictably high survival rates under masticatory forces (10-13).

The aim of this study was to assess the mechanical failure behavior of two types of porcelain-veneered crowns with metal core (IPS In-Line conventional feldespathic versus IPS In-Line press-on metal [PoM] [Ivoclar Vivadent, AG, Schaan, Liechtenstein]), when subjected to static compressive loading to the point of fracture, and to analyze fracture characteristics by scanning electron microscopy (SEM). The null hypothesis was that the pressed ceramic-to-metal (PoM) system would provide greater fracture resistance than the conventional metal ceramic system.

## Material and Methods

-Study design

The restorations used in this study were fabricated from a master cast, in the form of a maxillary molar of conventional shape, to obtain a full-coverage fixed crown. Forty-six impressions were taken from the master cast using addition silicone (polyvinyl siloxane) of heavy consistency and silicone fluid (Putty and Light Elite HD®, Zhermack, Italy) using the double-mix technique. Each impression was then cast in epoxy resin (Exakto-Form®, Bredent, Germany). After a 45-minute polymerization, each epoxy resin specimen was removed from the mold and mounted in a 22-mm-diameter copper cylinder, setting the specimen in type IV dental plaster (Pastel Rock Die Stone®, Kerr, Italy).

The specimens (n=46) were divided into two groups according to the veneering porcelain used: Group A – 23 metal-ceramic crowns with porcelain stratification layering (core: Rexillium V® nickel-chromium alloy, Pentron Laboratory Technologies; porcelain veneer: IPS InLine® ceramic, Ivoclar Vivadent); Group B – 23 metal-ceramic crowns with heat press ceramic (core: Rexillium V® nickel-chromium alloy, Pentron Laboratory Technologies, with porcelain veneer: IPS InLine PoM® ceramic, Ivoclar Vivadent).

The conventional veneer porcelain (IPS-InLine®) is in the form of a powder/liquid. Its standard composition is: (in wt%) SiO2 59.5 - 65.5, Al2O3 13.0 - 18.0, K2O 10.0 - 14.0, Na2O 4.0 - 8.0, Other oxides 0.0 - 4.0; pigments 0.0 - 2.0 and its coefficient of thermal expansion (CET) ranges from 12.60 to 13.20 ± 0.5 x 10-6 K-1.

The press-on veneer ceramic is in tablet form (ingots) (IPS InLine ® PoM). Its standard composition is: (in wt%) SiO2 50.0 - 65.0, Al2O3 8.0 - 20.0, Na2O 4.0 - 12.0, K2O 7.0 - 13.0, other oxides, fluoride 0.0 - 6.0; pigments 0.0 - 3.0, and its CET ranges from 13.0 to 13.3 ± 0.5 x 10-6 K-1.

-Crown morphology/design characteristics 

The design morphology of each crown followed the molar anatomy from which a wax-up was made. A silicon key was made from the wax-up, with axial thickness of 1mm and 1.5mm thickness on the occlusal aspect.

The internal crown cap was characterized by two inclined cuspal planes that allowed the porcelain veneer equal thickness over the entire crown surface. In the cervical area, the coping was precisely adjusted to the edge of the restoration piece.

The occlusal anatomy of each crown was designed using the wax-up technique, so that the load applicator of the Instron machine used for the compression tests (a 4-mm aluminum ball) made contact in the fossa of the restoration with three-point contact on the internal slopes of the vestibular cusps and palatine cusp.

Group A: 23 metal copings veneered with IPS-InLine conventional metal ceramics. The first and second dentin firing were carried out according to the manufacturer’s recommendations. Body porcelain was vibrated and condensed onto the copings. Firing used a Programat PX1 (Ivoclar Vivadent) furnace reaching a final temperature of 929ºC. The anatomy and thickness of the crowns were checked against the silicon key. Lastly, all specimens were finished and glazed conventionally.

Group B: 23 metal copings were veneered with IPS-InLine press-on metal (PoM) ceramic. A wax-up was built on the opaque metal frameworks using ash-free wax (XP Dent Corp., Miami, FL), checking anatomy and thickness. The wax-up was covered with plaster (Gilvest-HS, ICL Business Unit Materials.,Tel-Aviv, Israel) heated and cast (lost-wax technique) in a furnace (Jelrus Tremp-Mastre L two-stage., Buffalo, NY) placing the veneering cylinders and the porcelain ingots selected in the hot press furnace (Programat EP 500, Ivoclar Vivadent), which reached a final temperature of 1075ºC. The porcelain melts and is injected under pressure in the void left by the lost wax inside the veneering cylinder. Afterwards, the specimens were divested, finished, and glazed. This is a simpler and easier technique than that of conventional metal ceramics.

Once fabricated, the crowns were bonded using a dual-polymerization composite resin cement (Multilink® Ivoclar-Vivadent, Liechtenstein). Fatigue loading of each specimen was carried out with a chewing simulator (CS-4.4, thermocycling TC-3; SD Mechatronik, Feldkirchen-Westerham, Germany) producing a total of 120,000 masticatory cycles, with a vertical movement of 2mm, a frequency of 10 Hz, and a temperature range of 5-55ºCº. After fatigue loading simulation, the crowns were subjected to static loading until they fractured.

-Compression testing

Compression testing was carried out with a mechanical testing machine (Instron® model 4202, MA, USA). The load applicator descended onto the sample exercising continuous vertical force (5 KN load cell) with a crosshead speed of 0.5 mm/s, moving vertically downward perpendicular to the occlusal plane. The load force applicator’s aluminum ball established three-point contact with the internal slopes of the crown’s vestibular cusps and palatine cusp. The machine was stopped once the veneering ceramic had fractured, and the force that had provoked the fracture was measured in Newtons (N). Data were recorded with computer software.

-Statistical analysis

Statistical analysis was performed with SPSS for Windows (SPSS Inc., Chicago, IL, USA). Data were presented as variables of resistance, range, median, means and standard deviations (SD). The Chi-squared test was used to find out whether all the categories of a variable contained the same proportion. Student’s t-test was used for the comparison of means between the two veneering ceramics; for dichotomous variables, Fisher’s exact test was applied. The significance level was set at *p*<0.05.

-SEM analysis 

Firstly, all specimens were examined under an optical stereomicroscope (Leica APO MZ®, Leica Microsystems, IL, USA) with 8x and 12x enlargements to identify the type of fracture produced in each sample. When the first observation phase was complete, ten specimens were selected randomly, five from each group, to perform fractography analysis and then composition analysis using SEM. (JEOL JSM 6300 with crystal microanalysis Oxford Instruments Ltd, Tokyo, Japan).

## Results

Results are divided into:

Post-fatigue compressive testing results.

SEM analysis. 

1. Post-fatigue compressive test results

**Figure 1:**
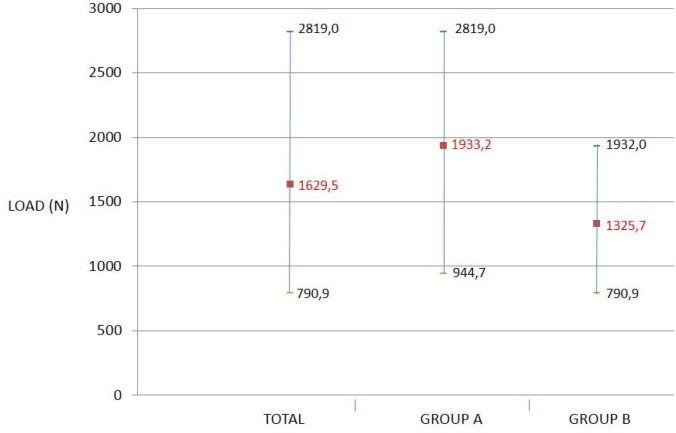
The difference between means of the tested groups A and B are represented. Group A (conventional metal ceramic) 45% higher than group B (pressed-on-metal ceramic) with a statistically significant difference.

1.a. Ceramic fracture resistance values:

Group A (conventional metal ceramic) obtained a mean fracture resistance value of 1933.17 N, while Group B (POM ceramic) obtained 1325.74N, with Group A values 45% higher than Group B (Table 1). Student’s t-test identified a statistically significant difference in fracture resistance between the groups (*p*=0.001) (Fig. 1).

**Table 1:**
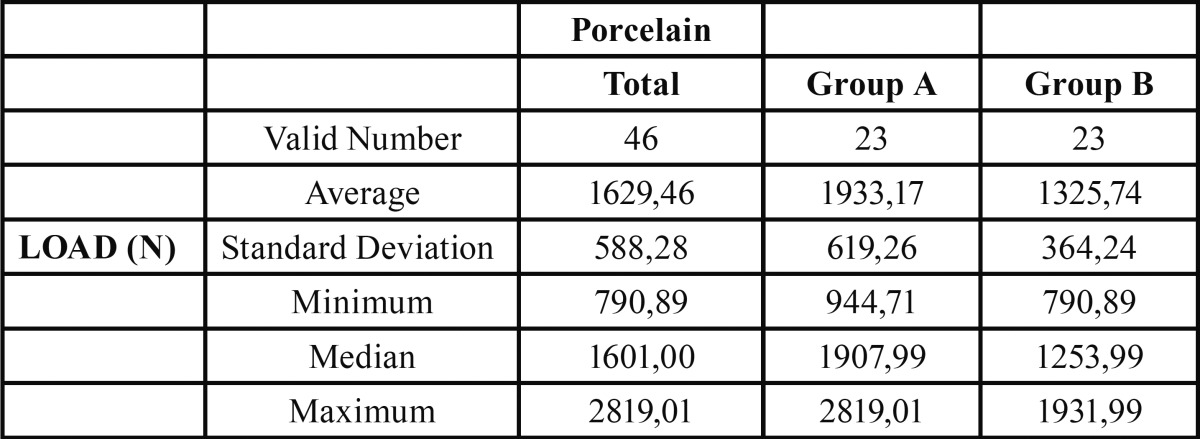
Descriptive analysis post-fatigue compressive testing results. Group A (conventional metal ceramic), group B (pressed-on-metal ceramic). Fracture resistance values in Newtons.

1.b. Fracture type 

The most commonly occurring type of fracture among the crowns was adhesive (n=38) ; only eight crowns suffered cohesive fracture, with statistically significant difference identified by the chi-squared test (*p*=0.00). However, comparing the two techniques by means of the Fisher test revealed a *p*-value of 0.500, and so no difference in fracture type.

2. SEM analysis 

**Figure 2:**
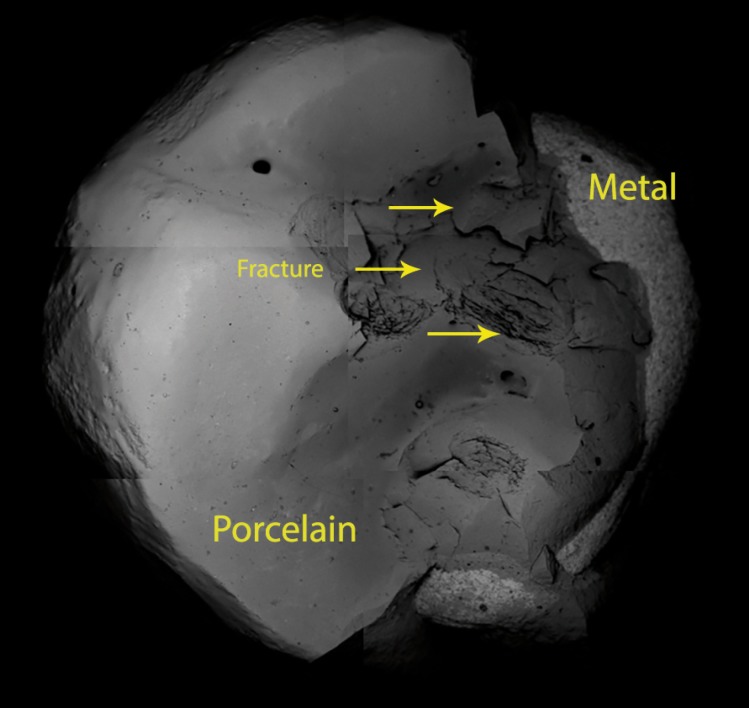
Type of adhesive fracture with metal exposure.

SEM microphotos show the crowns’ (occlusal) fracture zones and how fractures followed a radial or peripheral pattern; in other words, veneer porcelain deformation was produced in the occlusal area, producing a fracture of radial shape (Fig. 2).

**Figure 3:**
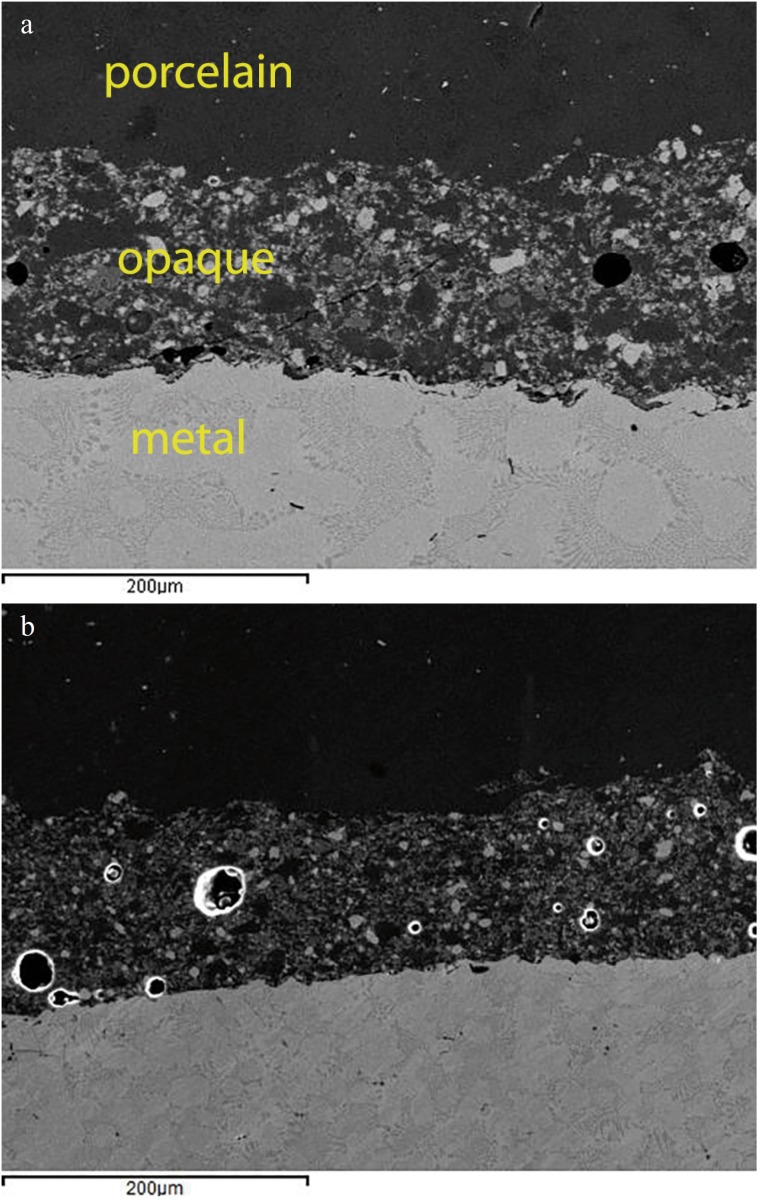
SEM 250x. a) Group A conventional metal ceramic. b) Group B pressed-on-metal ceramic. Microphotos revealed the characteristics of the different parts: the metal layer, the opaque layer (greater incidence of porosities in Group B and the surface porcelain.

SEM examination (250x magnification) of transversal sections of the crowns revealed the characteristics of the different parts in detail: the metal layer with its characteristic nickel texture, the opaque layer above (with bubbles visible in Group B) and the surface veneering porcelain (Fig. 3a,b).

**Figure 4:**
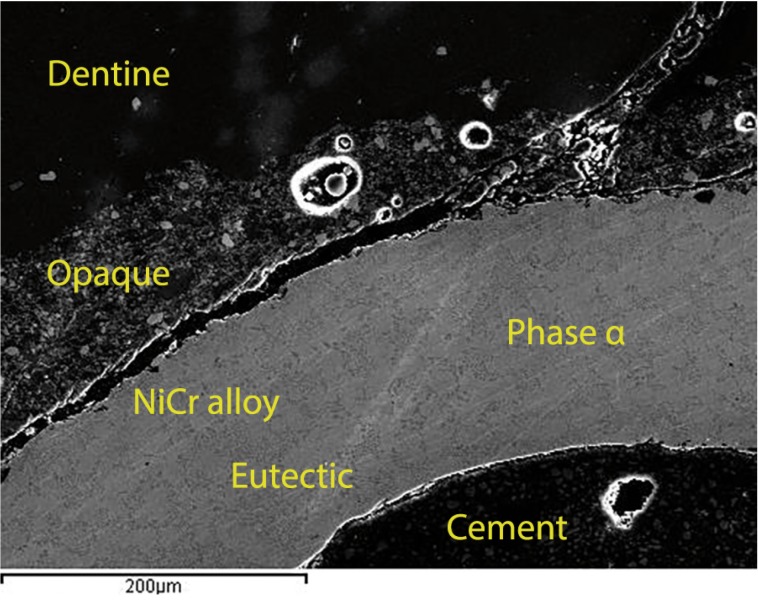
SEM 250x examination of transversal sections of a crown of group B. It reveals the characteristic path of an adhesive fracture (yellow arrow), from the porcelain surface towards the layer of opaque and from this layer to the metal, exposing the metal coping.

Fracture followed a course characteristic of adhesive fracture, from the surface porcelain towards the layer of opaque and from this layer to the metal, exposing the metal core (Fig. 4).

2.b SEM analysis of porcelain composition

When the compositions of the veneer porcelains in each test group were analyzed by SEM (Energy dispersive x-ray analysis at an electron voltage de 20 kV), both porcelains showed identical compositions, of the same elements with slight variations in percentages.

**Figure 5:**
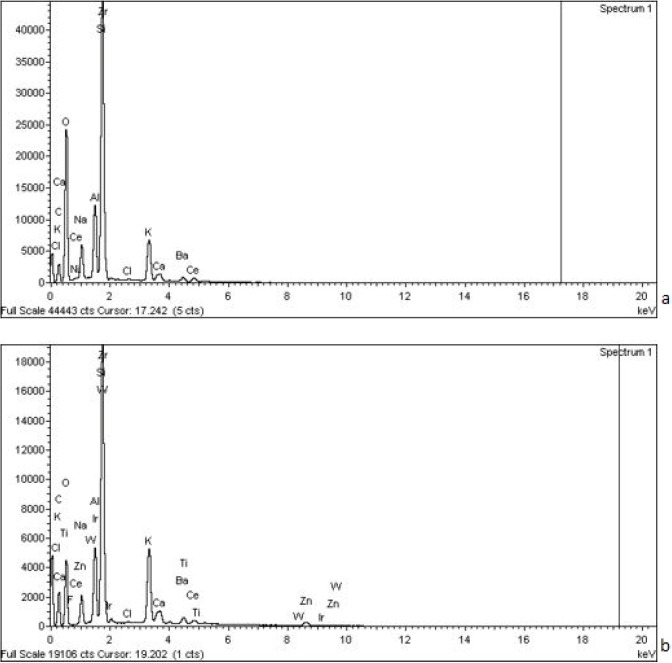
Chemical compositions of the veneer porcelains in each test group using SEM 500x. Values group A (a) and group B (b).

Mean values found were: SiO2 64.32, Al2O3 13.62, K2O 7.51, Na2O 7.70 in Group A, and SiO2 56.88, Al2O3 12.49, K2O 11.84, Na2O 4.95 in Group B (Fig. 5). These values are corroborated by the data supplied by the manufacturers (Ivoclar®).

## Discussion

-Methodology

The choice of strength test type and design – compressive testing in this case – was based on Clinical Research Associates (CRA) recommendations for studying the resistance of ceramic materials. 10 Other features of the study – study design, sample preparation, number of samples, loading speed, etc.– were similar to methods proposed by various other authors (12,13). According to the literature, pure compression testing would appear adequate for fracture resistance testing of crown and bridges (14-18). Before compression testing, samples were subjected to an ageing process as suggested by various authors (1,5,11).

Sample design in the present study took an upper first molar at 1:1 scale as real size offers results that are as close as possible to clinical reality, results were expressed as Newtons (N) (10,19,20).

-Results

The mean fracture resistance value for Group A conventional metal ceramic was 1933.17 N. Similar studies have also used real size metal-ceramic crowns and compression testing with the universal testing machine, obtaining results ranging from 1680N to 2335.16N (3,10,19-24).

The results obtained greater fracture resistance for the conventional metal ceramic samples (1933.17 N), while the PoM press-on metal ceramic obtained 1325.74 N. Student’s t-test identified a statistically significant difference in fracture resistance between the groups (*p*=0.001). In this way, the null hypothesis – that the pressed ceramic-to-metal (POM) system would provide greater fracture resistance than the conventional metal ceramic system – was rejected.

Other researchers have carried out similar studies with differing results. A study by Fahmy (21) obtained higher values with PoM, obtaining 2025.6N, while the conventional technique obtained 1810.3N.

Schweitzer (22) found no significant differences when comparing the bond strength of a pressed ceramic fused to metal compared with feldspathic porcelain fused to metal. Likewise, Venkatachalam *et al.* (4) made a comparative study of bond strength but did not observe significant differences in the mean fracture resistance of ceramic pressed to metal compared to feldspathic porcelain fused to metal.

The most commonly observed fracture types were adhesive fracture, a finding that coincides with studies by Alhasanyah (1) and Blatz (11). Agustín (10) also analyzed the mechanical behavior of four groups of crowns subjected to static loading – three types of zirconia crown and one metal-ceramic (IPS d.SIGN, Ivoclar Vivadent) – finding that 100% of the metal-ceramic crowns suffered adhesive fractures. However, Marker (19) observed a higher rater of cohesive fractures (under macroscopy).

-Scanning electron microscope analysis (SEM)

Konstantinos (24) studied the fracture resistance of metal-ceramic restorations, analyzing samples by SEM after loading, observing a radial fracture pattern around the loaded area. The present study observed the same radial or peripheral fracture pattern, which coincides with other authors’ observations (10,25,26).

The present study also noted a higher number of porosities and design imperfections in the porcelain veneers produced by the press-on technique (PoM) than with stratified ceramics, which might explain the different mechanical behavior observed in the study; Venkatachalam *et al.* (4) obtained similar findings under microscopy. In contrast to the study Drummond (7).

## Conclusions

The metal-ceramic crowns made with IPS InLine ceramics and IPS InLine PoM with different laboratory techniques achieved above-average values for clinical survival in the oral environment in accordance to ISO 6872.

Crowns made using the conventional IPS InLine technique showed 45% greater fracture resistance than IPS InLine PoM made with the press-on technique.

Veneer porcelain fractures in the occlusal contact area and the fracture displays a radial pattern.

The most common type of fracture is adhesive failure (with metal exposure).
